# Mechanobiology of Dental Pulp Cells

**DOI:** 10.3390/cells13050375

**Published:** 2024-02-21

**Authors:** Natalia Bryniarska-Kubiak, Agnieszka Basta-Kaim, Andrzej Kubiak

**Affiliations:** 1 Laboratory of Immunoendocrinology, Department of Experimental Neuroendocrinology, Maj Institute of Pharmacology, Polish Academy of Sciences, 12 Smętna St., 31-343 Kraków, Poland; basta@if-pan.krakow.pl; 2 Laboratory of Stem Cell Biology, Faculty of Biochemistry, Biophysics and Biotechnology, Jagiellonian University, 7 Gronostajowa St., 30-387 Kraków, Poland

**Keywords:** dental pulp cells, mesenchymal stromal cells, mechanobiology, mechanotransduction, stem cells, dental material, regenerative dentistry, tooth, biomaterials, biomechanics

## Abstract

The dental pulp is the inner part of the tooth responsible for properly functioning during its lifespan. Apart from the very big biological heterogeneity of dental cells, tooth microenvironments differ a lot in the context of mechanical properties—ranging from 5.5 kPa for dental pulp to around 100 GPa for dentin and enamel. This physical heterogeneity and complexity plays a key role in tooth physiology and in turn, is a great target for a variety of therapeutic approaches. First of all, physical mechanisms are crucial for the pain propagation process from the tooth surface to the nerves inside the dental pulp. On the other hand, the modulation of the physical environment affects the functioning of dental pulp cells and thus is important for regenerative medicine. In the present review, we describe the physiological significance of biomechanical processes in the physiology and pathology of dental pulp. Moreover, we couple those phenomena with recent advances in the fields of bioengineering and pharmacology aiming to control the functioning of dental pulp cells, reduce pain, and enhance the differentiation of dental cells into desired lineages. The reviewed literature shows great progress in the topic of bioengineering of dental pulp—although mainly in vitro. Apart from a few positions, it leaves a gap for necessary filling with studies providing the mechanisms of the mechanical control of dental pulp functioning in vivo.

## 1. Introduction

**Figure 1:**
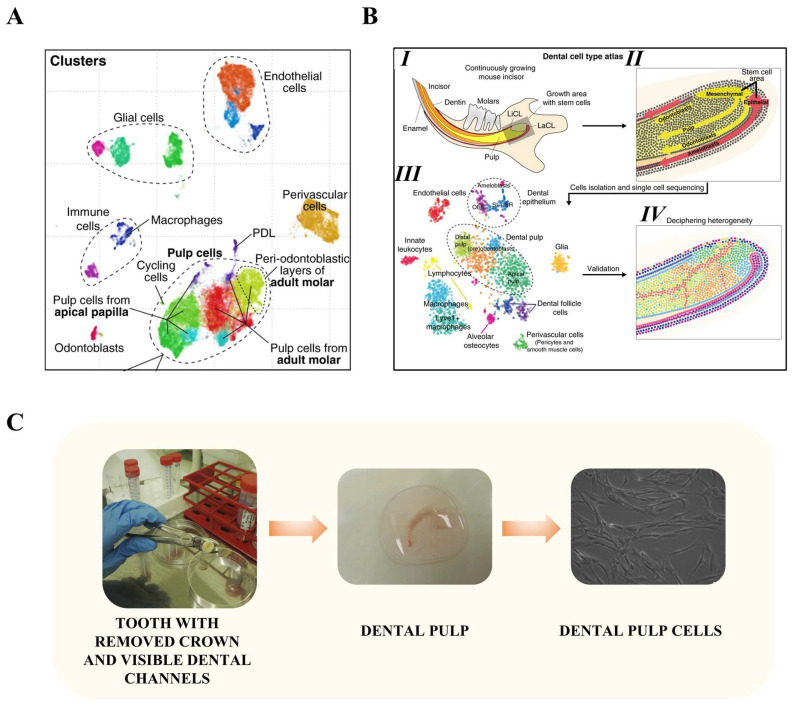
Heterogeneity of dental pulp cells and procedure of dental pulp cells isolation. (**A**) Heterogeneity of human dental pulp cells revealed by single-cell profiling of 39,095 cells. In total, 17 clusters were defined by Leiden clustering and are shown by different colors. The following populations are distinguished by the authors: endothelial cells, glial cells, immune cells including macrophages, odontoblasts, perivascular cells, periodontal ligament cells, pulp cells including cycling cells, pulp cells from the apical papilla, pulp cells from adult molar, peri-odontoblastic layers of adult molar, and periodontal ligament cells. (**B**) (**B-I)** Mice incisors are hypselodont teeth, which means that they grow continuously during mice’s lifespans. (**B-II**) Their growth is possible thanks to the existence of a stem cell niche in the apical pulp of the incisor. (**B-III**) PAGODA clustering revealed 17 cell subpopulations shown here in different colors. In mice, subpopulations consist of (**B-III**) endothelial cells, innate leukocytes, lymphocytes, macrophages, alveolar osteocytes, perivascular cells, smooth muscle cells, dental follicle cells, dental epithelium (ameloblasts, stratum intermedium cells, stellate reticulum cells, and outer enamel epithelium), and dental pulp cells (distal pulp cells, preodontoblasts, odontoblasts, and apical pulp cells). (**B-IV**) Those cell subpopulations were successfully mapped on the incisor, allowing identification of their niches. (**C**) Dental pulp can be isolated by exposing the tooth pulp using a dental drill and extracting the tooth pulp using a pulp extractor. Then, particular subpopulations including dental pulp stromal cells can be cultured and expanded in vitro. (**A**,**B**) Modified and reprinted with permission from Krivanek et al. [1] (**C**) and Labedz-Maslowska A., Bryniarska N., Kubiak A., et al. [13]. These works are licensed under a Creative Commons Attribution 4.0 International License. To view a copy of this license, visit http://creativecommons.org/licenses/by/4.0/, accessed on 24 October 2023).

The dental pulp is the inner part of the tooth responsible for properly functioning during its lifespan. Together with other dental cells, it creates a very heterogeneous environment maintaining homeostasis in the tooth and tissue around it. In humans, dental cells include endothelial cells, glial cells, perivascular cells, immune cells, odontoblasts, periodontal ligament cells, neurons, and dental pulp cells [1,2] (Figure 1A). The latter, also considered as dental pulp stem/stromal cells (DPSC) (Figure 1C), were firstly described by Stan Gronthos et al. in 2000 [3]. In this pioneering work, the authors describe them as the counterparts of mesenchymal stromal cells derived from bone marrow residing in tooth roots. By immunocytochemistry, staining DPSCs were shown to express similar markers like bone marrow stromal cells (BMSCs), including but not limited to Integrin-β1, Collagen I, Collagen III, Osteonectin, Alkaline Phosphatase, and others. At the same time, the authors showed that DPSCs contain more colony-forming cells and more actively proliferating cells than BMSCs. Finally, after in vivo transplantation into immunocompromised mice, DPSCs formed a dental structure consisting of dentin, odontoblast, and pulp-like tissue with blood vessels set in layers corresponding to the histological organization of teeth [3]. Multipotent mesenchymal stem cells exist in the hypselodont tooth (in animals with continuously growing teeth, i.e., horse, mouse, rat) (Figure 1B), while in the brachydont tooth, (i.e., human) the existence of such a niche ceases to exist after the final formation of teeth roots. Nevertheless, even in adult dental tissue some pulp cells retain their stem cell properties and can be activated upon injury to support tissue regeneration [4]. Dental tissues are known to originate from ectoderm—and more precisely cranial neural crest cells [5]. In pioneering work, Kaukua et al. (2014) showed that Sox10+/PLP1+ peripheral glia cells in mice give rise to dental mesenchymal stem/stromal cells which are then capable of differentiating into both pulp cells and odontoblasts. Those findings raised interest in the use of DPSCs in the context of neural tissue regeneration. It results in several papers describing more or less advanced neuronal differentiation of DPSCs [6,7,8,9,10]. In some of those studies, DPSCs undergoing neurogenic differentiation partially exhibit features of electrical activity characteristic for neurons [10]; in other studies, the success of differentiation was claimed mostly due to the expression of proteins characteristic for neurons [6,7]. Such an observation should, rather, lead to subdued enthusiasm for using unmodified DPSCs as a potential source of neurons since, similarly to other stromal cells, their capability for differentiation is rather limited to the tissue of origin—dental tissue [11]. At the same time, it is widely known and recognized that regenerative dentistry is strongly associated with the use of multiple biomaterials characterized by particular biomechanical properties [12].

Since stromal cell differentiation strongly relies on the biomechanical properties of the niche [14], in the present work we review the mechanobiology of dental pulp cells. We address how mechanical cues impact dental pulp physiological and pathological states and ultimately how knowledge about dental pulp mechanobiology might be utilized in the context of regenerative medicine.

## 2. Mechanical Cues in the Physiology and Pathology of Dental Pulp

### 2.1. Biomechanics of the Dental Pulp and Surrounding Tissues

**Figure 2:**
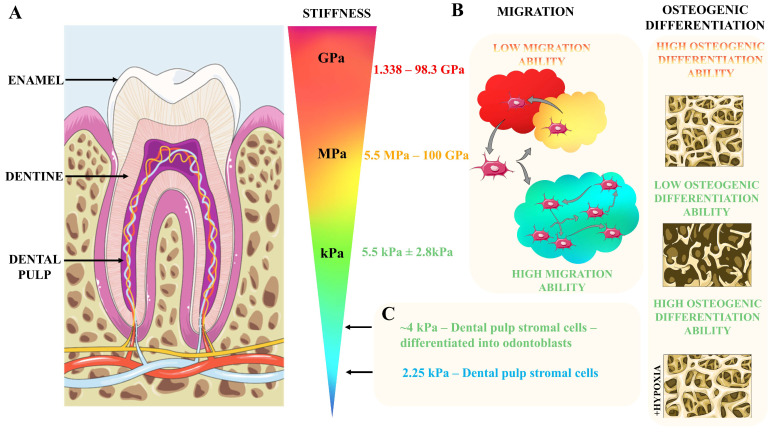
Mechanical properties of teeth compartments and their impact on dental cells. (**A**) Schematic drawing of human teeth with literature data about mechanical properties of its major components. The outermost layer of teeth, enamel, elastic moduli range from 1.338 to 98.3 GPa [19,20,21,22,23,24], the intermediate layer of teeth, dentine, elastic moduli range from 5.5 MPa to 100 GPa [19,20,21,22,23,25,26,27,28,29,30], while the innermost dental pulp elastic moduli were reported to be 5.5 kPa [32]. (**B**) Impact of mechanical functionality like migration velocity and osteogenic differentiation of dental pulp cells in vitro based on [13,34,44]. (**C**) Mechanical properties of dental pulp stromal cells cultured in the control conditions and after induction of odontoblastic differentiation based on [33].

**Figure 3:**
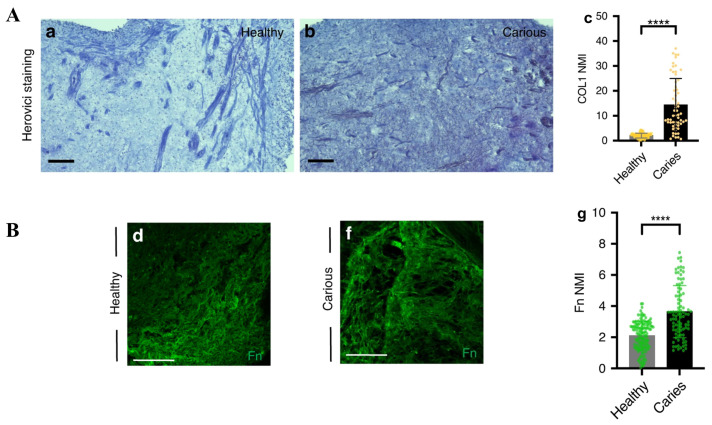
Impact of caries on extracellular matrix composition of dental pulp. (**A**) Expression of collagen in the human dental pulp of (**A-a**) control teeth and (**A-b**) carious teeth revealed by Herovici staining. (**A-c**) Normalized mean intensity quantification proving increased expression of collagen in various dental pulp. **** *p* < 0.0001. (**B**) Expression of fibronectin in human dental pulp of (**B-d**) control teeth and (**B-f**) carious teeth revealed by immunofluorescent staining. (**B-g**) Normalized mean intensity quantification proving increased expression of fibronectin in carious dental pulp. **** *p* < 0.0001. (Modified and reprinted with permission from Balic et al. [35] This work is licensed under a Creative Commons Attribution 4.0 International License. To view a copy of this license, visit http://creativecommons.org/licenses/by/4.0/, accessed on 23 December 2023).

Organism development strongly relies on multiple biological pathways and processes, nevertheless, recent years have provided strong evidence of the crucial role of physical interaction in this vital process [15]. As mentioned, mesenchymal stem cells responsible for continuous growth on the murine tooth originate from neural crest cells [5,16]. During development, neural crest cells exhibit durotaxis—migration in a gradient of stiffness from a softer to stiffer environment [17]. Consequently, neural crest cell differentiation is stiffness-dependent. Human neural crest stem cells differentiate into Schwan cells when cultured on hydrogel with a stiffness of 15 kPa. On the other hand, culturing on stiff hydrogel with a stiffness of 1 GP (1000 kPa) resulted in the promotion of differentiation into smooth muscle cells [18]. The mechano-sensitivity of neural crest cells might be treated as a premise for the mechanical modulation of neural crest to achieve the desired differentiation outcome—also including obtaining dental pulp cells. Significantly, teeth as tissue are very heterogeneous in the context of their mechanical properties. The outer teeth part, the enamel, is characterized by a very high Young modulus in the range of 1.338 to 98.3 GPa [19,20,21,22,23,24] (Figure 2A), depending on the method used to determine Young’s modulus as well as the tooth source. In the case of dentine, the intermediate part of teeth, variability in Young’s moduli values was significantly higher, and depending on the report ranges between 5.5 MPa and 100 GPa [19,20,21,22,23,25,26,27,28,29,30] (Figure 2A). Nevertheless, those values are among the highest in the context of Young’s moduli of all tissue and thus makes the tooth one of the stiffest compartments in the organism. Importantly, the hardness of the tooth is strongly associated with proper angiogenesis inside the dental pulp. Mice with a knockout of *Vegfr2* genes specific for endothelial cells were characterized with a tooth hardness decreased to 1 N in comparison to 4 N in control animals. The authors of the work provided a model of interactions between odontoblasts and endothelial cells leading to extensive angiogenesis and vessels sprouting around odontoblasts with the parallel maturation of odontoblasts mediated by endothelial cells. Angiogenesis impaired in mice with *Vegfr2* knockout leads to insufficient transport of phosphatase—crucial in the process of dentinogenesis [31]. This study shows the complexity of interactions between particular teeth cells and compartments in the principle significance of the proper functioning of dental pulp cells for overall teeth performance. While dentine and enamel Young’s modulus is in the range of MPa to GPa, the dental pulp is ultimately a softer compartment with mean Young’s modulus of 5.5 ± 2.8 kPa (what is 5.5 × 10^−6^ GPa) [32] (Figure 2A). Correspondingly, atomic force microscopy characterization revealed that a Young’s modulus for DPSCs of 2.25 kPa increases significantly during odontoblastic differentiation. An increased stiffness of DPSCs undergoing odontoblastic differentiation was associated with actin reorganization characterized by more pronounced stress fibers [33] (Figure 2C). Also, the DPSCs’ ability to migrate is correlated with the biomechanical features of their environment. Ehlingera et al. [34] compared the migratory capability of DPSCs on PDMS substrates of varying stiffness, in the range from 1.5 kPa to 2.5 MPa to glass characterized by a Young’s modulus of several GPa reflecting enamel stiffness. The authors observed that DPSCs migrate with the highest velocity on the softest substrate, and velocity decreases gradually with increasing substrate stiffness. In the same study, the authors showed that DPSCs migrate with the highest velocity on glass coated with laminin, intermediate velocity on collagen, and the lowest velocity on fibronectin-coated glass [34] (Figure 2B). Those findings show that not only the stiffness of the niche but also its molecular composition affects the ability of DPSCs to migrate. Thus, tuning those features might be crucial in the proper design of biomaterials for tooth regeneration, especially because extracellular matrix (ECM) secretion is strongly affected by the tooth’s condition. Single-cell RNA sequencing revealed that carious teeth dental pulp cells differ significantly in the expression profile of ECM proteins in comparison to intact teeth. In the case of dental pulp fibroblast, a four-fold increase in the number of Osteoglycin-expressing cells in the carious dental pulp was reported by the authors. Similarly, the percentage of fibroblast subpopulations expressing Integrin Binding Sialoprotein (a marker of osteoblast/odontoblast differentiation) was increased 22.5 fold times in carious dental pulp fibroblasts. Consequently, in a cluster of mesenchymal stromal cells, the authors observed a more than two-fold increase in cells expressing ECM molecules including *MCMAM*, *MYH9*, *FN1*, and *COL14A1*. Importantly, those scRNAseq data were supported by the immunohistochemical analysis of protein expression in the dental pulp. Collagen 1 and Fibronectin 1 fluorescence intensity was significantly higher in dental pulp histological section from carious teeth than in healthy ones (Figure 3). Finally, the authors of this work noticed a significant increase in the expression of multiple interleukins (*IL-2*, *IL-3*, *IL-4, IL-13*, *IL-17A*, *IL-17F*, *IL-21*, *IL-23A*, and *IL-26*) in the immune cells clusters of carious dental pulp [35]. This last observation is strongly in line with several studies showing that enhanced immune activation correlates with altered ECM composition and biomechanical properties of the tissue as reported for neuroinflammation in the course of an ex vivo ischemic stroke model [36], the swelling of lymphatic nodes upon mouse immunization by keyhole limpet hemocyanin [37], and an in vitro model of diabetic retinopathy, in which the treatment of endothelial cells with TNF-α and IL-1β induces changes in the expression of laminin β1 and fibronectin [38]. In turn, the treatment of dental pulp stromal cells with TNF-α increases the expression of integrin α6 and consequently leads to enhanced DPSCs migration in both the transwell assay and scratch assay. Consequently, the treatment of DPSCs with siRNA targeting integrin α6 decreased the migration of DPSCs [39]. Several other chemotactic molecules have been shown to play an important role in the mobilization of DPSCs in injured dental tissue including HGF, FGF-2, TGFβ-1, CCL2, CXCL14, CXCL12, G-CSF, S1P, and C5a (reviewed with details: Rombouts et al. [40]). Under such conditions, various metalloproteinases are overexpressed in injured dental tissue (MMP-1, MMP-3, MMP-7, MMP-9, MMP-13) [41]. Interestingly, MMP-3 overexpression is associated with regenerative processes within injured dental pulp by supporting angiogenesis and dentin formation [42]. Finally, ECM alone might act as a factor promoting DPSCs performance in various biological assays significant from the point of view of regenerative medicine. Lee et al. showed that demineralized dentine matrix enhances DPSCs expansion, the deposition of mineralized matrix, and, in parallel, decreases the expression of apoptotic markers in DPSCs in vitro. On the other hand, the authors of this work showed that supplementation with DDM does not affect the migration of DPSCs into collagen hydrogel [43]. Thus, the proper modulation of ECM composition and dental pulp mechanics might be a promising approach to tuning immune reactions in the context of dental tissue regeneration after the removal of caries and the accompanying inflammation.

### 2.2. Biomechanical Cues in Dental Pain Propagation

Dental pulp inflammation is one of the key biological processes strongly related to the development of caries and other tooth diseases and pathologies. Nevertheless, from the patient’s perspective, dental pain associated with a pathological state is a strong burden. Dental pain is one of the most common sources of pain in the population; it might be caused by damage to hard dental tissue like enamel, dentin, or cementum or in more severe cases by dental pulpitis. In most cases, this type of pain is considered to be acute pain caused by the propagation of mechanical signals through dental tissue to nerves localized in the dental pulp [45]. Before pulpitis is developed, dental pain might be felt due to an impaired composition of dentine. In multiple cases, the reason for such pain is associated with either cold or hot temperature to which the tooth is exposed. In general, dentine is hard tissue fenestrated by multiple dentinal tubules [46]. In intact teeth, dentinal tubules are filled with dentinal fluid, and the processes of odontoblasts are localized on the border of dentine and dental pulp. Stress factors applied to the tooth lead to the movement of dentinal fluid and, in consequence, activate nociceptors in dental pulp by the application of shear stress. One such pain-evoking factor is the feeling of pain caused by hot and cold. Lin et al. [47] propose a model in which both the changes in the radius of the dentinal tubule and dentinal tubule expansion/contraction contributed to dentinal fluid flow and in turn to pain sensation in dental pulp by affecting mechanosensitive ion channels in neuronal cells. The inward flow of the dentinal fluid causes pain related to hot which lasts longer and is characterized by lower intensity. An outward flow of dentinal fluid causes pain induced by the cold which lasts for a short period and is more severe. Importantly, the authors emphasize that mechanosensitive receptors rather than thermo-sensitive receptors are responsible for feeling pain in the context of cold stimulation—this is because the time of below 1 s after a neuronal discharge is observed is shorter than the time needed to cool down the dental pulp and induce a response from the thermo-sensitive receptors inside the dental pulp. In the context of pain resulting from hot stimulation, the authors do not exclude that, together with the mechanical activation of cells in dental pulp due to the inward flow of fluid in dentinal tubules, thermo-activated ions channels might be engaged in this process because pain sensation is characterized by a longer latency (>10 s) than in case of cold pain [47]. Such a mathematical model, developed on a single dentinal tubule [47], is in line with in vivo experimentation performed on humans, aiming to determine the relationship between fluid flow through dentine and the severity of pain. In this study, the dentine of an intact tooth designed for extraction for orthodontic reasons was exposed to flow applied both outwards and inwards. The severity of pain experienced by patients was assessed by a visual analog scale (VAS). The authors observed that for negative pressure stimuli (outwards flow), the median pain threshold was 125 mmHg, while for positive pressure stimuli (inward flow) the median pain threshold was 200 mmHg. Since the pain threshold indicates the moment of significant pain feeling in this study, outwards flow was shown to induce severe pain earlier than inward flow. Correspondingly, for the same absolute values of pressure, more severe pain was felt for outward flow than for inward flow [48]. The aforementioned mechanism applies when dentin is exposed to thermal/physical stimuli and could occur due to various defects in enamel and dentine. In the majority of cases, a complete cure for this type of pain is possible and could be achieved either by changing eating habits or the use of desensitizers (i.e., toothpaste containing potassium salts) [49]. On the other hand, pulpitis, caused either by caries or mechanical damage of the tooth, is also manifested by dental pain, which in this case might be continuous and very bothersome for the patient [50,51]. In the course of pulpitis, the inflammatory response leads to an increased permeability of blood vessels, extravasation, and in turn, increased pressure inside dental pulp—which is covered by stiff dentine and enamel. Such pressure might stimulate C-fibers in dental pulp leading to dull pain [52]. In 1983, Tønder and Kvinnsland [53] showed that interstitial fluid pressure in the inflamed dental pulp of cats (16.3 ± 2.8 mmHg) is significantly higher than in the untreated dental pulp of cats (5.5 ± 0.95 mmHg) [53]. A physical mechanism based on increased pressure in dental pulp leading to the excitation of dental pulp nerves is accepted [54]; to the best of our knowledge, the precise mechanism of this process, taking into account physical factors together with the mechanosensitivity of neurons, is still missing. In any case, severe pulpitis and associated pain usually lead to the final treatment, which is a tooth extraction or root canal treatment. In the first case, the outcome is the loss of the whole tooth, while in the second the outcome is the loss of the whole dental pulp. Since multiple patients are bothered with both of those issues there is a need for further research aiming to provide new therapeutic strategies in regenerative dentistry based on the bioengineering of dental pulp.

## 3. Importance of Mechanical Cues in Bioengineering of Dental Pulp and Tooth

Current regenerative dentistry is mainly focused on the use of implants and bone reconstruction [55,56]. On the other hand, despite multiple existing preclinical models and initial outcomes proving safety in the use of DPSCs in the clinic, a clinically validated strategy allowing for the reconstruction of human dental pulp after it is lost for a variety of reasons, including root channel treatment, is missing.

### 3.1. Mechanical Factors in Dental Pulp Regeneration

**Figure 4:**
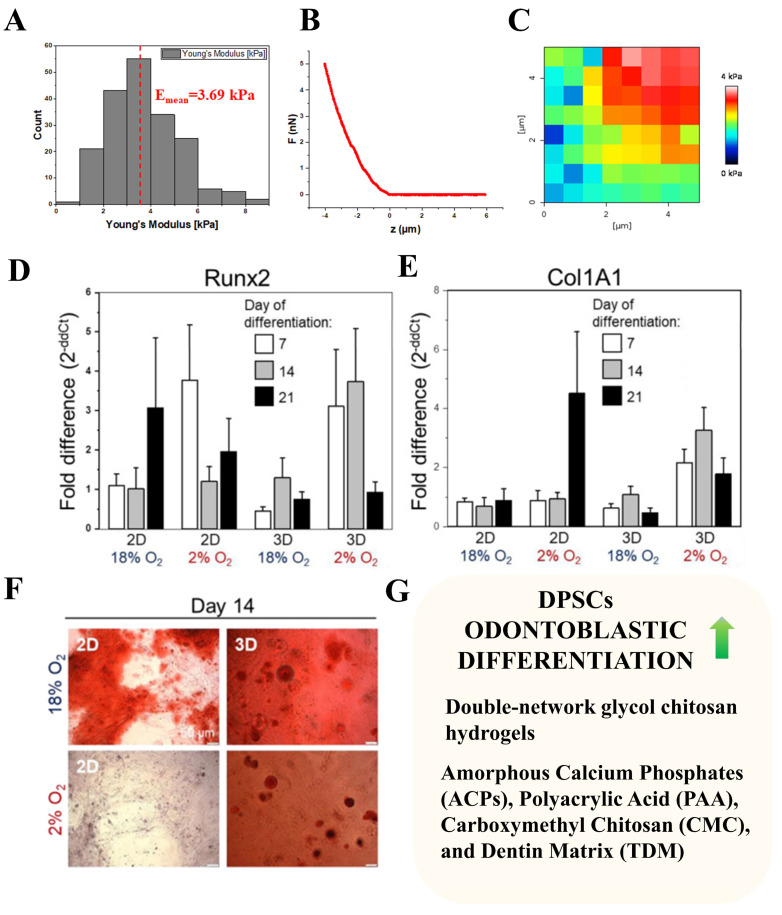
Impact of encapsulation of dental pulp stromal cells on their osteogenic and odontogenic differentiation. (**A**) Distribution of elastic moduli of BD PuraMatrix hydrogel determined with the use of atomic force microscopy. (**B**) Exemplary force curve recorded on BD PuraMatrix hydrogel with the use of atomic force microscopy. (**C**) Elasticity map revealing mechanical heterogeneity of BD PuraMatrix hydrogel. (**D**) Expression of mRNA for osteogenic transcription factor *Runx2* in human dental pulp stromal cells undergoing osteogenic differentiation in the following conditions: 2D vs. 3D (BD PuraMatrix hydrogel encapsulated cells), 18% O2 vs. 2% O2 atmosphere. (**E**) Expression of mRNA for the osteogenic differentiation marker extracellular matrix protein *Col1A1*, condition of differentiation the same as in (**D**). (**F**) Degree of mineralized matrix deposition by dental pulp stromal cells osteogenic differentiation for 14 days for the same conditions as in (**B**). (**G**) Encapsulation of dental pulp stromal cells with hydrogels characterized by specific chemistry also enhances their odontoblastic differentiation—based on [57,61]. (Modified and reprinted with permission from Labedz-Maslowska A., Bryniarska N., Kubiak A., et al. [13] This work is licensed under a Creative Commons Attribution 4.0 International License. To view a copy of this license, visit http://creativecommons.org/licenses/by/4.0/, accessed on 23 December 2023).

One of the crucial parameters describing the mechanical properties of a cell niche is its stiffness. As mentioned above, the tooth is very heterogeneous in the context of its mechanical properties, with a Young’s modulus ranging from several kPa for dental pulp [32] to hundreds of GPa for enamel/dentine. While the majority of biomaterials designed for implants are characterized by a Young’s modulus in the range of GPa [55], such very high stiffness is far away from the physiological mechanics of dental pulp. DPSCs cultured on PAA hydrogels coated with fibronectin exhibit a stiffness-dependent capability to differentiate into osteogenic lineages. By assessing the deposition of the mineralized matrix, alkaline phosphatase activity, and expression of Osteopontin, the authors claimed that osteogenic differentiation was the most pronounced on stiff glass rather than on PAA hydrogels (Figure 2B) [44]. On the other hand, DPSCs encapsulated inside a three-dimensional peptide hydrogel (BD PuraMatrix) with a stiffness of ~3.5 kPa outperformed DPSCs cultured on two-dimensional glass in their osteogenic differentiation, but only in conditions of hypoxia (2% of O_2_). Those results indicate that not only the stiffness of the cell niche is important for the differentiation of DPSCs, but also how cells interact with the niche (two-dimensional vs. three-dimensional) (Figure 2A and Figure 4A–F) [13]. While those studies aimed to assess the osteogenic differentiation of DPSCs, it seems that the most promising strategy with the use of DPSCs is rebuilding dental pulp—especially in the context of root channel treatment. In recent work, Han et al. [57] address this challenge by the generation of three-dimensional hydrogels with DPSCs encapsulated inside and injecting them into a model system of root channels. Authors compare single-network and double-network glycol chitosan hydrogels. They observed that a double-network hydrogel possesses biomechanical properties closer to the physiological dental pulp and supports the odontogenic differentiation of DPSCs (Figure 4G) [57] Very promising results in the context of dental pulp regeneration were provided in a study by Itoh et al. The authors generated three-dimensional constructs filled with human DPSCs. Those three-dimensional constructs were successfully maintained in cell culture for up to 21 days. To test its potential clinical application, the authors established an in vivo assay in which human pulpless teeth were transplanted subcutaneously in immunodeficient mice after filling their empty root channel with DPSCs-constructs. Six weeks after transplantation, dental pulp-like tissue was rich in blood vessels and also contained odontoblast-like cells on the boundary with dentine. This study shows a very promising example of how the proper three-dimensional cell culture treatment of DPSCs might contribute to their regenerative capacity in vivo [58]. Also, the generation of external forces on DPSCs was shown to enhance their odontogenic differentiation. DPSCs seeded on biomimetic substrates fenestrated to mimic dentinal tubules, were characterized by more pronounced odontoblastic differentiation while compressed with a pressure of 19.6 kPa [59]. Dynamic hydrostatic pressure (HSP) applied to primary human DPSCs also strongly affected DPSCs differentiation. DPSCs subjected to HSP decreased their number and adhesion, they differentiated into the osteogenic and odontoblastic lineage. HSP-subjected cells deposited more mineralized matrix and odontoblastic marker DSPP in response to BMS-2 stimuli [60]. A recent study by Wen et al. [61] indicates that the use of hydrogels composed of Amorphous Calcium Phosphates (ACPs), Polyacrylic Acid (PAA), Carboxymethyl Chitosan (CMC), and Dentin Matrix (TDM) increases DPSCs differentiation towards odontoblastic and odontogenic lineages. PAA-CMC-TDM hydrogel was also used in the context of the repair of damaged dental pulp in dogs, where it showed the ability to induce the repair of dentine similar to the commercially available ceramic I Root BP. Moreover, the authors assessed the aforementioned hydrogels’ in vivo potential for cranial and femur bone regeneration in rats. PAA-CMC-TDM hydrogels promoted the regeneration of both femurs and cranial bones. Importantly, the authors checked the biosafety of PAA-CMC-TDM by assessing the histological heart, liver, and kidney and did not observe any significant anomalies in these organs while comparing to control groups (Figure 4G) [61]. The osteogenic potential of DPSCs was also shown when DPSCs were cultured on a Biocoral scaffold (porous hydroxyapatite) [62]. Woloszyk et al. [63] showed that DPSCs cultured on silk fibroin scaffolds in osteogenic medium and spinner flask bioreactor lead to a 6–7 fold time decrease in the expression of tooth-associated markers (*Nestin* and *Dentin Sialophosphoprotein*) with a corresponding almost 2-fold increase in the expression of osteogenic markers (*Osteocalcin* and *Collagen type I*). Thus, the authors stated that the proper mechanical and biological stimulation of DPSCs might shift DPSCs toward osteogenic differentiation [63]. In another study, DPSCs exhibited osteogenic potential [13,44,64] (Figure 2A and Figure 4); on one hand, this creates a potential for using DPSCs in the regeneration of bone defects (especially in context cranial bones derived from ectoderm [61,63]), but on the other hand, it generates challenges in the regeneration of functional dental pulp. In principle, it is crucial to find a way of handling DPSCs in a way that inhibits their differentiation into osteogenic lines, while those cells are destined for dental pulp regeneration. In principle, the existence of calcification within dental pulp is considered a negative event during dental pulp regeneration [61].

### 3.2. Biomechanical Cues in the Regeneration of Vasculature and Nervous Fibers within the Dental Pulp

Another significant challenge associated with dental pulp regeneration is the regeneration of dental pulp vessels and neuronal networks. Human DPSCs cultured on silk fibroin scaffolds exhibited proangiogenic capabilities when tested in the chicken embryo CAM model [65]. Very promising results in the context of dental pulp regeneration were obtained by Siddiqui et al. in their work on angiogenic peptide hydrogel utilized for root channel regeneration. In their work, they used injectable, acellular SLan peptide hydrogel. SLan hydrogel was injected into the canine dental channel after the removal of the whole dental pulp. The authors observed pronounced angiogenesis and the existence of neuronal bundles in teeth filled with SLan hydrogel. Since before the injection of hydrogels, all dental pulp was removed and root channels were widened and thoroughly cleaned, it is very important to understand the mechanism of hydrogel colonization by a variety of dental pulp cells. The potential migration of dental pulp stem cells from the apical papilla is excluded because the authors properly chose the canine model—so a model of an animal with continuously growing teeth and consequently no stem cells in the apical papilla. Nevertheless, at this point, the authors have not provided the mechanism of regeneration in SLan which would be crucial before moving this promising strategy toward clinical practice [66]. More mechanistic insight into the proangiogenic capabilities of DPSCs was provided by a study depicting the co-culture of DPSCs with HUVECs cells in BD PuraMatrix peptide hydrogel. The authors observed that the co-culture of those cells encapsulated in hydrogel enhances capillary formation by HUVECs, affecting HUVECs migration and increasing VEGF secretion. Interestingly, such co-culture was also available to induce the odontogenic differentiation of DPSCs [67], which is in line with another observation that in mice odontoblasts guide vessel sprouting as well as endothelial cells, which promote proper odontoblasts functioning [31].

While dental pulp stem/stromal cells and their progeny, odontoblasts, are shown to induce angiogenesis [31], neurogenesis within regenerated dental pulp appears to be a more challenging process. As mentioned above, several reports indicated that DPSCs might be differentiated into neural-like cells. Nevertheless, the neuronal functionality of those neural-like cells remains questionable and should be treated with big precautions. For a strategy in which bulk dental pulp cells are transplanted in a biocompatible carrier into teeth root, it could be hypothesized that neurons remaining in the bulk dental pulp cell population will survive the process of dental pulp processing. At the same time, several reports indicated that the neurogenic potential of DPSCs is associated with their paracrine activity [68,69,70]. In principle, it was shown that DPSCs secreted multiple growth factors associated with nerve growth and regeneration: nerve growth factor (NGF), brain-derived neurotrophic factor (BDNF), neurotrophin-3 (NT-3), and glial cell line-derived neurotrophic factor (GDNF) [69,70]. Such findings might be partially explained by the fact that stromal cells, in general, aim to maintain the niche in which they reside by secreting soluble factors (i.e., multiple subpopulations of stromal cells support hematopoietic stem cells in bone marrow [71,72]). DPSCs in comparison to BMSCs migrate more robustly into neurodegenerative injury sites when tested in vitro. Both stromal cell populations became activated while exposed to conditioned media from injured neurons. Stromal cell activation results in an increased expression of proteins associated with homing including SDF-1α, CXCR4, VCAM-1, VLA-4, CD44, and MMP-2 [73]. While compared to BMSCs, DPSCs exhibit a higher expression of neurotrophic factors: NGF, GDNF, and NT-3 both at levels of mRNA and protein. A lack of differences in the expression of BDNF between those two populations of stromal cells at both protein and mRNA levels was observed. Those differences were reflected in the results of a functional test of axon outgrowth from trigeminal ganglia (TGG) and dorsal root ganglia (DRG). In both cases, the co-culture of neuronal cells together with DPSCs resulted in more pronounced axonal growth than in the case of co-culture together with BMSCs [74]. A great example of paracrine activity in functional human dental pulp was shown by Mitsiadis et al. [75] in their work about nerve growth factor signaling in the dental pulp. The authors showed that, in intact dental pulp, NGF and its low-affinity receptor p75NTR expression is restricted to nerve fibers in the dental pulp. However, upon injury, the expression of NGF and its high-affinity receptor TrkA is increased in odontoblasts in the injury site of teeth [75]. Thus, we suggest that research on DPSCs in the context of their neurotrophic properties rather than direct neuronal differentiation could be especially beneficial in the context of dental tissue regeneration. The establishment of dental pulp cell handling protocols and culture that allow neurons from dental pulp to survive could be challenging, but in any case, might provide a great breakthrough in the context of dental pulp regeneration. Neurons are mechanosensitive cells [76]. Neurite outgrowth [77,78] and neural stem cell differentiation [79,80] are dependent on tissue stiffness with soft substrates promoting neurite outgrowth and neuronal differentiation. Because the dental pulp is stiffer than brain tissue [32,81], a proper tuning of hydrogel used for dental pulp repairment might be challenging, especially in the initial period of dental pulp regeneration, when we predict that neurite outgrowth occurs to the biggest extent. One of the approaches that might be beneficial in this context is the modulation of mechanosensitive channels, Piezo1/2, which play an important role both in neuronal [82], as well as in dental, cells [83].

### 3.3. Mechanosensitive Receptors Piezo1 and Piezo2 and Dental Pulp

Piezo1 and Piezo2 are mechanosensitive calcium ion channels described by the Ardem Patapoutian group, which was awarded with Nobel Prize in Medicine and Physiology in 2021 [84]. Importantly, Piezo1 possesses its modulators, agonists Yoda1, and Jedi1/2, and antagonists, Dooku1 and GsMTx4. The use of that molecule can affect a variety of neuronal cell functions including neurite outgrowing, neural stem cell differentiation, and the course of neurodegenerative diseases (reviewed here: [82]). Consequently, Piezo1 and Piezo2 play important roles in the functioning of dental pulp cells. Stem cells from human exfoliated deciduous teeth (SHED) odontogenic differentiation was enhanced when cells were cultured under hydrostatic pressure. Hydrostatic pressure led to the activation of the Wnt/βcatenin signaling pathway in a manner dependent on the activity of the mechanosensitive Piezo1 channel [85]. Similarly, in DPSC-derived cells, SDP11, hydrostatic pressure leads to a Piezo1-dependent enhancement of osteogenic differentiation and subsequent inhibition of adipogenic differentiation [86]. Piezo1 activation by its agonist, Yoda1, promoted the migration of DPSCs. This effect was inhibited by the use of Piezo1 antagonists: ruthenium red or GsMTx4 [87]. Yoda1 modulation of Piezo1 was also reported to promote the osteogenic differentiation of human dental follicle cells (hDFCs). The authors observed an increased expression of osteogenic lineage markers ALP, RUNX2, OCN, and BMP2 at both mRNA and protein levels. In the same study, the authors observed that the modulation of Piezo1 with Yoda leads to an enhanced proliferation of hDFCs [88]. The proliferation of DPSCs isolated from rats’ incisors was also increased when low-intensity pulsed ultrasound (LIPUS) led to the activation of Piezo1 in DPSCs [89].

While the aforementioned works mainly contribute to the role of Piezo1 in the context of regeneration, it is important to address the role of Piezo1 and Piezo2 in sensory events within the tooth. Both Piezo1 and Piezo2 are expressed in odontoblasts [90,91]. The mechanical stimulation of odontoblasts leads to the activation of Piezo1, resulting in increased intracellular Ca^2+^ concentration. In the same study, the authors observed that the pharmacological stimulation of Piezo1 with its agonist, Yoda1, led to a decrease in mineralized matrix deposition, while an shRNA-mediated knockdown of Piezo1 enhanced mineralized matrix deposition by odontoblasts.

Taking these results together shows that mechanical stimulation recognized by Piezo1 affects the functioning of odontoblasts and thus might play a role in the transduction of physical signals from dentinal tubules [90]. Such a hypothesis seems to be possible because Piezo1 is abundant not only in odontoblast bodies but also in their projections—which are considered to sense mechanical signals from dentinal tubules. Consequently, shear stress leads to an increased concentration of calcium ions inside odontoblasts [92]. Piezo1 is also expressed in nerve fibers within dental pulp. In the case of fibers, its expression in A was shown in 60.2% of small myelinated axons (Aδ) and 24.3% of large myelinated axons (Aβ), while in the case of fibers C its expression was noticed in 15.5% of unmyelinated axons [93]. Neurons engaged in signal transmission from dental pulp also express the Piezo2 mechanosensitive receptor. Its expression was reported in medium and large-size dental primary afferent neurons (DPA neurons) building myelinated fibers A. Functionally, those neurons exhibit RI current upon mechanical stimulation. Importantly, those currents were undetectable after the blockage of Piezo2 with ruthenium red, but also while Piezo2 was knocked down by siRNA [94].

Taking all those observations together, the abundant expression of Piezo1 and Piezo2 in dental pulp neurons makes them a promising target for pharmacological intervention in the case of dental pain. Because recent years brought very intensive focus in research on Piezo1 and Piezo2 receptor modulation, data about their modulator’s pharmacology should be also available, allowing us to assess whether those molecules might be used in pharmacological interventions in dentistry.

### 3.4. Molecular Pathways Regulating DPSCs Mechanosensitivity

It should be underlined that, apart from Piezo1 and Piezo2, other ion channel receptors are involved in mechanosensitivity—including acid-sensing ion channels (ASICs). While ASICs were primarily associated with pH sensing, recent studies suggest that mechanical stimuli also activate ASIC1, ASIC2, and ASIC3 channels. Numerous mechanosensitive ion channels are expressed in odontoblasts (TRPV1, TRPV2, TRPV4, TRPC1, TRPC6, TRPP1, TRPP2, TRPM7, TRPM3, TREK1) and DPA neurons (TRPA1, TRPV1, TRPV2, TRPV4, TRPM3, TRPM7) [95,96]. At the same time, several other proteins play a role in DPSCs mechanosensitivity. Among them, YAP/TAZ and MAPK are associated with DPSCs differentiation, while Rho GTPases and Wnt are associated with DPSCs proliferation [97].

YAP/TAZ is a transcription coactivator whose functionality depends on its localization within cellular compartments. Cytoplasmic localization is associated with dampened YAP/TAZ activity while nuclear localization allows for YAP/TAZ binding to transcription sites and interaction with transcription factors including TEADs. The cytoplasmatic localization of YAP/TAZ is controlled by mechanical stimuli including substrate stiffness, cellular shape, or physical cell-cell interactions leading to contact inhibition [97]. In dental pulp cells, a static magnetic field induces the nuclear localization of YAP/TAZ proteins leading to an enhanced expression of *CTGF* and *ANKRD* as well as enhanced proliferation of DPSCs. Consequently, the mechanism of nuclear translocation of YAP/TAZ depends on the actin cytoskeleton since the treatment of cells with Cytochalasin D leads to a decreased nuclear translocation of YAP/TAZ in DPSCs subjected to static magnetic field [98].

YAP/TAZ nuclear translocation promotes the odontoblastic and osteogenic differentiation of DPSCs. DPSCs cultured on a PLGA scaffold spontaneously differentiated into osteogenic/odontoblastic lineage. DPSCs growing on the closed side of the PLGA scaffold exhibited spread morphology, robust stress fibers, a high level of nuclear YAP, and an enhanced level of osteogenic markers *COL1A1* and *ALP*. DPSCs differentiation was hampered by the use of verteporfin—YAP transcriptional inhibitor. As a result, both the nuclear localization of YAP and the expression level of *COL1A1* and *ALP* decreased [99].

Further study revealed that the PLGA-induced osteogenic differentiation of DPSCs is mediated by the YAP/beta-catenin signaling axis. In turn, this suggests a potential interplay between YAP and Wnt in the context of DPSCs [100]. The wnt/β-catenin pathway is also crucial for DPSCs proliferation. PDMS substrate stiffness regulates the proliferation rate of DPSCs. DPSCs proliferated at the lowest rate on the softest PDMS, while the rate of DPSCs proliferation was the highest on the stiffest PDMS substrate. In the same work, the authors observed that both beta-catenin expressions were gradually increasing with an increasing stiffness of PDMS, while the expression level of the beta-catenin negative regulator, GSK-3beta, was gradually decreasing with an increasing stiffness of PDMS. Finally, the authors of this work showed that the osteogenic differentiation of DPSCs is also stiffness dependent and the expression of several osteogenic differentiation markers (*ALP*, *OPN*, *OCN*, *BMP-2*, *Runx-2*) increases gradually with increasing PDMS stiffness [101]. Interestingly, lipopolysaccharide (LPS) stimulation enhanced the odontoblastic differentiation of DPSCs. The treatment of LPS-subjected DPSCs with MAPK inhibitor led to an inhibition of DPSCs odontoblastic differentiation [102]. MAPK phosphorylation was also elevated in DPSCs in which odontoblastic differentiation was achieved by a mechanical compression of DPSCs in 3D scaffolds [59]. Summarizing, several molecules and pathways with well-known modulators are involved in the mechanosensitivity of DPSCs. This creates changes in DPSCs modulation for both basic research aiming to understand dental pulp mechanobiology and also in the context of dental pulp engineering.

## 4. Concluding Remarks and Future Directions

From the moment DPSCs were described by Gronthos et al. [3], DPSCs have been under extensive research interest in the context of tissue regeneration, with particular interest in dental tissue regeneration. While biological factors play a significant role in the regeneration of all tissue, recent years have provided more evidence that biomechanical factors are crucial for success in regenerative medicine. In this context, we reviewed recent and past literature to depict the topic of dental pulp cell mechanobiology. A lot of work in this field has been done so far, mainly in in vitro systems. On the one hand, this research is very important, because it provides the principle mechanisms of dental pulp functioning in the context of physical stimulation [59,89,90], hydrogel encapsulation [13,57,67], and substrate stiffness sensing [34,44]. On the other hand, fewer in vivo studies have been conducted so far. Often, studies performed with the use of animal models focus on testing hydrogels or cells injected into various models of teeth defects rather than depicting mechanisms of the mechanical control of various processes in the dental pulp. In the present work, we referred to recent work describing changes in extracellular matrix composition within dental pulp upon inflammation [35] and also the significance of the interplay between odontoblasts and endothelial cells for teeth mineralization [31]. However, we see a need for more mechanistic research aiming to understand how physical factors contribute to tooth development as well as pain propagation—especially in the process of pulpitis. This research will provide important knowledge that will allow for finding new therapeutic strategies in the treatment of most bothersome dental diseases. The significance of mechanobiology is proven in various other research fields including developmental biology [103,104,105], immunology [106,107,108], cancer treatment, and stem cell biology. Mechanical factors play a key role in cancer development and progression [109]. Knowledge about mechanobiology and anticancer drug action [110,111,112] is leading not only to a better understanding of drug action on cancer cells but also to the design of more efficient platforms for anticancer drug screening [113]. At the same time, knowledge about the stiffening of bone marrow during aging [114] leads to new strategies for hematopoietic stem cell rejuvenation based on the use of hydrogels with tuned stiffness [115]. Those examples show that even basic research addressing the mechanobiology of various biological systems could lead to significant discoveries contributing to medical applications.
